# Fukushima Health Study Launched

**DOI:** 10.1289/ehp.119-a428a

**Published:** 2011-10-01

**Authors:** Winifred A. Bird

**Affiliations:** Winifred A. Bird is a freelance journalist living in Nagano, Japan. Her work has appeared in the *Japan Times*, *Science*, *Yale Environment 360*, *Dwell*, and other publications.

How does exposure to low-level radiation affect a person’s risk of developing cancer or other health problems? Is there a threshold below which there is no effect at all? Can we really understand the effects of prolonged low-level exposure by applying what we’ve learned from one-time blasts like the Hiroshima and Nagasaki atomic bombs?

Following the reactor fuel meltdowns at Japan’s Fukushima Daiichi nuclear power plant in the spring of 2011, these unsolved puzzles—long the subject of debate among radiation epidemiologists^1^^,^^2^—have taken on deep personal importance for millions of Japanese potentially exposed to radioactive fallout from the damaged plant.^3^ In June Fukushima’s prefectural government launched an ambitious project aimed, in part, at providing some answers—although experts warn the accident’s health impacts may not become clear for decades, if ever.^4^^,^^5^

Study leader Seiji Yasumura, an epidemiologist at Fukushima Medical University, says the main goal of the Fukushima Residents’ Health Management Survey is improved health care for those exposed to radiation through ongoing free health screenings and provision of other services should those screenings identify problems. Some residents will also receive mental health questionnaires and related services. “If we put research first, people wonder if they’re being used as laboratory animals, and that’s not our intention,” he says.^6^ But Yasumura and colleagues also intend to analyze the data they collect for evidence regarding the health effects of low-level radiation.

The researchers plan to combine activity logs for each of the prefecture’s 2 million–plus residents with maps plotting daily radiation levels to estimate the external radiation dose each person may have received in the first four months after the crisis began March 11. Questionnaires distributed to three towns in late June and prefecture-wide starting in late August ask residents to recall their whereabouts and activities during the period following the still-unresolved crisis.^7^ The survey requires extremely detailed, hour-by-hour activity information through March 25. For the period after that, respondents are asked to fill in their weekly routine, including hours spent outside at specific locations, as well as any major departures from that routine.

This method, which also was used after the 1986 Chornobyl nuclear power plant disaster,^8^ hinges on the ability of study subjects to remember their activities, hour by hour, after a gap of several months. This raises questions about the reliability of the exposure estimates. The problem is not unique: For both logistical and political reasons, past survey-based studies of radiation exposure have often taken years to get off the ground.^9^ “[Given that Japanese officials] have also had to deal with the accident, having questionnaires out in five months is amazing,” says John Boice, scientific director of U.S.-based International Epidemiology Institute and a professor of medicine at Vanderbilt University.

The researchers plan to combine activity logs for each of the prefecture’s 2 million–plus residents with maps plotting daily radiation levels to estimate the external radiation dose each person may have received in the first four months after the crisis began March 11.

There are other complications in estimating exposure for the people of Fukushima. The time lag between the most severe exposure and the start of the study means researchers cannot physically check internal levels of iodine-131. Iodine-131 exposure is a concern in Fukushima because the quick-to-decay radionuclide caused thyroid cancer in thousands of people who, as children, drank contaminated milk following the Chornobyl accident.^10^ Although the Fukushima project will include free life-long thyroid cancer screening for all 360,000 residents who were under 18 years of age at the time of the accident, and the health of all residents will be tracked for at least 30 years, data on internal iodine-131 exposure exist only for 1,150 children tested in mid-March.^11^^,^^12^^,^^13^ Kagoshima University cancer epidemiologist Suminori Akiba says that won’t be enough to determine the relationship between future thyroid cancer rates and radiation from Fukushima Daiichi.

Steve Wing, an epidemiologist at the University of North Carolina at Chapel Hill who studied cancer incidence following the 1979 Three Mile Island nuclear accident,^14^ says a host of other factors could throw off dose estimates and lead to flawed conclusions later on as well. For instance, radiation-level maps will be constructed using extensive monitoring data from the central, prefectural, and local governments, and from independent organizations. However, a spokesperson at the prefecture’s project management office says data gaps exist, and Wing points out that levels can vary even within small plots because of variations in weather and in the natural and built environment.

External exposure experienced after the time period upon which the survey focuses could also impact overall exposure, as could internal exposure from food, water, and air (the survey includes questions about drinking water sources and consumption of home-grown food, but Yasumura says the resulting data will be used only to single out potential high-exposure individuals for further health checks). “The key goal for an unbiased [cancer] study is to place people into exposure groups accurately. If people are mixed up, any association would be diluted,” Wing says.

In later phases of the study, researchers will need to track the shifting lifestyles and living environments of the huge study cohort, then disentangle the often-powerful impact of those factors from that of radiation.^15^^,^^16^ Japan’s comprehensive system of official family and residence registries, along with nationwide mortality data and regional cancer registries, will likely make the task more feasible, says Kiyohiko Mabuchi, head of the Chernobyl Research Unit at the National Cancer Institute. Given the tremendous uncertainties entailed in each stage of the study, Wolfgang Hoffmann, a professor at the University of Greifswald’s Institute for Community Medicine in Germany, says the project is unlikely to provide a definitive answer on dose–response relationships or resolve knotty questions such as whether childhood leukemia incidence increases with proximity to nuclear power plants. “There are better ways to study those things,” Hoffman says. “The Fukushima study should focus on the best medical care for the Fukushima residents rather than [seeking] to resolve controversial issues in radiobiology.”

According to Boice, assuming current estimates of radionuclide releases and dose–response models are correct, the doses received by most Fukushima residents are likely to be so low as to render any related increase in cancer occurrence undetectably small, even with 2 million people in the study. But Mabuchi says the project is still essential in terms of both public health and science: “All accidents are different. We don’t know enough to predict what will happen in Fukushima. The only way to find out is to do an actual study.”
